# GPURFSCREEN: a GPU based virtual screening tool using random forest classifier

**DOI:** 10.1186/s13321-016-0124-8

**Published:** 2016-03-01

**Authors:** P. B. Jayaraj, Mathias K. Ajay, M. Nufail, G. Gopakumar, U. C. A. Jaleel

**Affiliations:** Department of Computer Science and Engineering, National Institute of Technology Calicut, NITC Campus, Calicut, Kerala 673601 India; Center for Cheminformatics, Open Source Pharma, No. 22, World Trade Centre, Malleswaram, Bengaluru, Karnataka 560055 India

**Keywords:** *In-silico* drug discovery, Virtual screening, Ligand based drug discovery, Random forest classifier, GPU computing, CUDA

## Abstract

****Background**:**

*In-silico* methods are an integral part of modern drug discovery paradigm. Virtual screening, an *in-silico* method, is used to refine data models and reduce the chemical space on which wet lab experiments need to be performed. Virtual screening of a ligand data model requires large scale computations, making it a highly time consuming task. This process can be speeded up by implementing parallelized algorithms on a Graphical Processing Unit (GPU).

****Results**:**

Random Forest is a robust classification algorithm that can be employed in the virtual screening. A ligand based virtual screening tool (GPURFSCREEN) that uses random forests on GPU systems has been proposed and evaluated in this paper. This tool produces optimized results at a lower execution time for large bioassay data sets. The quality of results produced by our tool on GPU is same as that on a regular serial environment.

****Conclusion**:**

Considering the magnitude of data to be screened, the parallelized virtual screening has a significantly lower running time at high throughput. The proposed parallel tool outperforms its serial counterpart by successfully screening billions of molecules in training and prediction phases.

## Background

Conventional drug design relied on in-vitro methods. In these methods, wet lab experiments are employed to discover the activity of ligands with a target protein molecule. Depending on this approach to determine the activities of a large set of molecules would consume a lot of time and money. *In-silico* drug design breaks this bottleneck by using modern computational techniques. *In-silico* approach increases the speed and reduces the cost of drug design. Depending upon the structural knowledge of the ligand and proteins, there are four different approaches used in *in-silico* drug discovery. They are structure based drug design, ligand based drug design, de-novo design and library design. The most widely used approaches are structure based drug design and ligand based drug design [1].

Ligand based drug design works by building a conceptual model of the target protein. Ligand based virtual screening uses this model to evaluate and separate active molecules for a target protein. This process involves the application of classification algorithms on the conceptual model. The model acts as the training data [2, 3] for the classification algorithms used in virtual screening. These algorithms are expected to learn the model parameters of the input training data. After the training phase, these algorithms are applied on molecules whose activities with the target protein are unknown. Based on the properties of the training data, the algorithms will be able to classify these new molecules as active or inactive. This eliminates wet lab experiments involving inactive molecules from being carried out [4]. Hence, this approach drastically reduces the number of molecules with which the activity of a target is to be studied.

Implementing virtual screening using sequential computing techniques often fail to produce results within a reasonable time frame. The complexity of operations exhibited by virtual screening encourages the usage of parallel computing techniques to tackle them. But, the installation and maintenance of an infrastructure for parallel computing with a large number of processors, such as a Multiple Instruction Multiple Data (MIMD) system, is neither cost effective nor energy efficient. Computing on Graphical Processing Unit (GPU) offers a large number of computational cores that are capable of performing floating point computations in parallel. The computation performance obtained by running this algorithm on a GPU is comparable to that of a CPU cluster [5]. A GPU infrastructure can be installed and maintained at a comparatively low cost. Hence, the advantage of using GPU computing is twofold; it is both cost effective and energy efficient. Therefore, implementing a parallelized version of virtual screening using GPU computing is highly desirable.

The aim of virtual screening is to filter inactive molecules from the active ones and not to misclassify any active molecules. The success of ligand based drug discovery depends on the effectiveness of the classifier used in virtual screening. Ideal virtual screening requires a classifier that could produce no false negatives and a lower number of false positives. This results a reduction in the spectrum of wet lab experiments being conducted. This reduces the cost. The popular classification algorithms that are widely used for virtual screening are Support Vector Machines (SVM) [6], Random Forest Classifiers (RFC) [7, 8] and Navïe Bayes Classifiers [9]. This is because they have been found useful in many domains of bioinformatics and medicinal chemistry [10–12].

### Random forest classifier

Random forest classifiers work by growing a predetermined number of decision trees simultaneously [13]. The internal nodes of a decision tree may contain one or more values. Each such internal node represents possible outcomes of the problem given to it. Splitting of internal nodes is based on maximum information gain using GINI index. The growth of the tree is restricted either by pruning or by setting a threshold value on the splitting criteria. The classification process is as follows. A test instance is run on all the decision trees grown in the forest. Each tree’s classification of the test instance is recorded as a vote. The votes from all trees are aggregated and the test instance is assigned to the class that receives the maximum vote.

### Parallel decision trees

There are many reported works in this area. Some of them are outlined in this section.

Nuno Amado et al. [14] presents an overview on the various ideas regarding implementing parallel decision trees using data parallelism, task parallelism and hybrid parallelism in their work. They also describe a new parallel implementation of the C4.5 decision tree construction algorithm using breadth first strategy. A method [15] for implementing the evaluation and training of decision trees and forests implemented completely on a GPU (non-CUDA version) was reported in 2009. A ubiquitous parallel computing approach for constructing a decision tree on GPU is also available [16]. This work exploits the divide and conquer parallelism in ID3 at two levels. One at the outer level of building the tree node by node in a top-down fashion. And the other at the inner level of sorting data records within a single node level. Grahn et al. presents a parallel version of the Random Forest machine learning algorithm namely CudaRF [17] which was implemented using the Compute Unified Device Architecture (CUDA). In this implementation, one CUDA thread is used to build one tree in the forest. The forest is constructed on the GPU using one thread for each decision tree in the random forest.

The study in [18] compares the effectiveness of Field Programmable Gate Arrays (FGPAs), multi-core CPUs and GPUs for accelerating classification using models generated by Compact Random Forest (CRF) machine learning classifiers. It was noted that FPGAs provided the best performance and performance per watt. Multi-core CPUs with OpenMP based implementation ensured scalability. GPUs offered the best performance per dollar and more than twice the performance of CPUs. The issue with GPUs is that the performance deteriorated with larger classifiers.

All previous works [15–17] to use GPUs for Random Forest classification have relied on coarse-grained task parallelism and have yielded unsatisfactory results. Their attempts seem to underutilize the available parallelism of graphics hardware and have undergone only cursory evaluations. Liao et al. introduced CudaTree [19], a GPU Random Forest implementation which adaptively switches between data and task parallelism.

## Implementation

The motivation for the parallelization of the process of building a random forest stems from the fact that decision trees in a random forest are built independent of one another. This results in the faster construction of decision trees. Thus, deployment of parallelized random forest speeds up the ligand classification during the virtual screening phase.

**Fig. 1:**
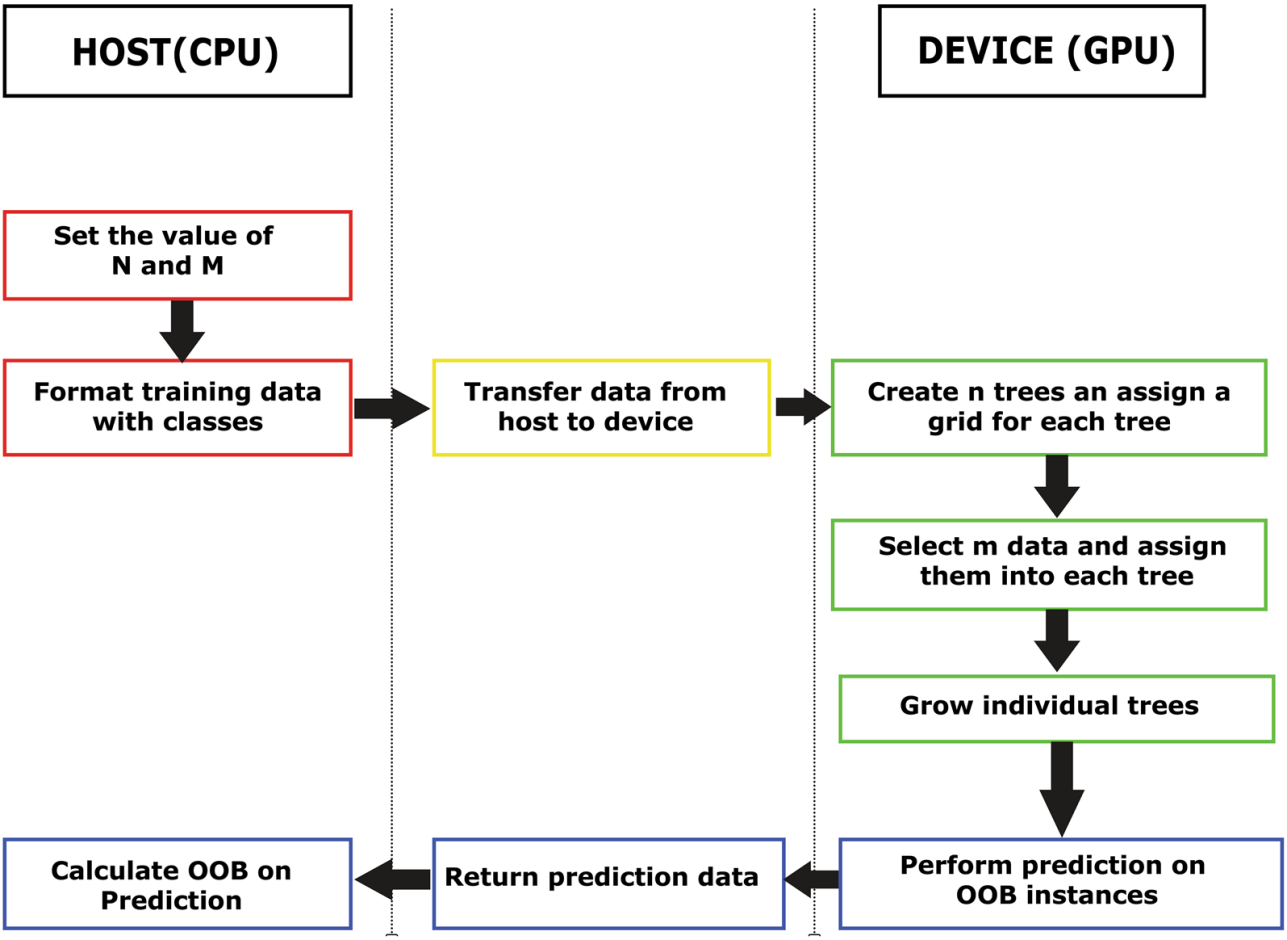
CPU–GPU execution control flow in the proposed parallel algorithm

Figure 1 illustrates the execution flow and communication between the host and the device for the execution of the proposed tool.

In GPU based computing, the process of growing each tree is assigned to a set of cores. The samples and features of a given tree are determined from the host system. The decision tree is built level by level on a grid, by assigning a block to build a level in a tree. The device memory of the GPU is used to manage the tree data in a grid. Each grid performs its evaluation independently.

Building a random forest requires growing a certain number of decision trees. Many decision tree algorithms are based on recursion. But, the kernels executed on the GPU do not support recursion [20]. So, the use of recursion is not feasible in the GPU based Random Forest algorithm. This necessitates the design of an iterative tree building algorithm. The data and output of each such decision tree are not interdependent until execution is completed. Hence, each such decision tree can be handled as an independent thread in the GPU. There are other parallel decision tree learning algorithms [19, 21, 22] that work by clubbing data and task parallelism. This is similar to the approach taken in the proposed algorithm.

There are two approaches to parallelize the building phase of a decision tree, namely data parallel depth first tree construction and fine grained task parallel breadth first construction [19]. The depth first tree construction uses the GPU to compute the optimal split point for a single node of the decision tree. Each CUDA thread block is responsible for a subset of samples from a single feature, followed by a parallel evaluation of the optimal feature split thresholds. In breadth first tree construction, instead of constructing a single tree node a whole level of tree nodes is created simultaneously.

In the serial implementation of Random Forest, the depth first method is adopted to split the nodes based on selected features. From the GPU perspective, the depth first tree construction method will become more expensive as the tree grows deeper. This is because of a large number of kernels created to perform feature split on a relatively small number of samples. Similarly, the breadth first construction is less efficient at the top of a tree, as the kernels would need to perform task parallel threads on less number of nodes.

In order to completely utilize the parallelism provided by the GPU, a hybrid approach is adopted in the proposed algorithm. The decision tree on GPU is constructed, starting from the root node in a depth first manner. After a certain point, the tree construction is switched to breadth first approach. This crossover point determines the efficiency of the GPU implementation and the effectiveness of the results produced by the Random Forest. This crossover point can be determined by setting a threshold on the number of nodes that is present in the decision tree. Once, the number of nodes in a decision tree crosses this threshold, the tree construction strategy is switched from depth first to breadth first. A very low value of this threshold will result in poor performance of the algorithm whereas a very large value will make the tree building slower. The only possible way to create a decision tree in a depth first manner in the GPU environment is by using a stack. The breadth first method of tree construction uses a queue. It is computationally faster to use the breadth first method for large data on a GPU. So it is more productive to use this hybrid method for constructing decision trees on a GPU.

### Determining the breadth first crossover threshold

The instance at which point the size of the sub-tree is large enough to merit a switch from depth first to breadth first tree construction is critical. The scikit-learn [23] serial algorithm uses the depth first method for the tree construction. In the proposed parallel implementation, calculating and fixing the crossover threshold determines the speed of the algorithm. Since the number of features used in the present implementation is fixed for the parallelization of a given tree, the techniques derived by Liao et al. [19] are used here also. The optimal value of the crossover threshold is derived by varying the number of samples, features, and classes used in the classification. The optimal crossover value obtained through regression technique is:

$$3705 + 0.0577 * n + 21.84 * f$$

where *n* is the number of samples and *f* is the number of features considered at each node split. Therefore, the optimal crossover point is dependent on the number of features and the number of training samples used in the training process.

## Proposed parallel algorithm

Random forest building on a GPU begins with a parallelized bootstrapping of the input data items on CUDA as shown in the Algorithm 1. It is then followed by the parallel creation of decision trees in depth first manner. GINI index is used for splitting the data. The *childArray* is used as a stack to create nodes as described in the Algorithm 1. After the number of nodes in the decision tree crosses the threshold, the tree creation switches to breadth first mode. Here, the *childArray* works as a queue and creates a set of nodes level by level as shown in Algorithm 1. The algorithm terminates by eliminating those nodes that fail to attain a threshold GINI index value.
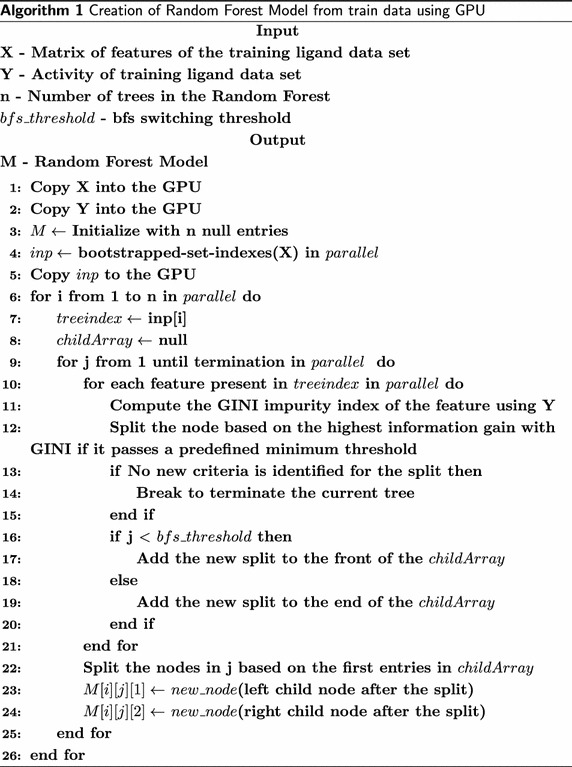

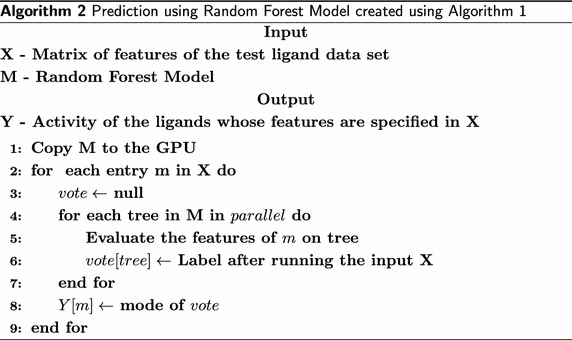


Random forest prediction run on a GPU is shown in Algorithm 2. To classify a new instance, it is classified by all the trees in the forest in parallel. Each tree evaluates the features for the new instance. The label after processing the input is recorded as a vote. The votes from all trees are combined and the class for which maximum votes are counted (majority voting) is declared as the class of the new instance.

## Results and discussion

The computational performance and quality of results obtained from the GPURFSCREEN for ligand based drug discovery are presented in this section.

### Datasets used

Datasets with different numbers of molecules were used to gain insight into the quality of the result produced by this GPU version. Training data sets were obtained from NCBI PubChem [24] bioassay database which was prepared from frozen stocks of Mtb H37Rv obtained from American Type culture collections. For the screen, amikacin was included in the positive control wells in every assay plate. The bio-assay SDF file downloaded from PubChem was supplied as input to the PowerMV/CDK [25–28] feature extraction tool to generate 2D molecular descriptors. A total of 179 descriptors were generated for each dataset of the experiment. The selection of descriptors was based on the criteria that they are sufficient to characterize the drug-likeness of a compound [4]. These descriptors fall into three categories. The first eight descriptors are used mainly to characterize the drug-likeness of a compound. Another set of twenty-four continuous descriptors considered are based on a variation of BCUT descriptors to define a low dimensional chemistry space. The last 147 bit-string structural descriptors, known as Pharmacophore Fingerprints, are based on bioisosteric principles. The dataset used for training are shown in Table 1. The dataset for testing is taken from GDB17 [29], a chemical universal database for unknown compounds that has been enumerated by Lars Ruddigkeit et al.

**Table 1:** Datasets used

PubChem bioassay datasets	Number of molecules	Date of access
AID 1332	1193	22/07/2014
AID 492952	2294	22/07/2014
AID 651616	5569	22/07/2014
AID 2330	36,869	22/07/2014
AID 893	68,532	24/09/2014
AID 778	95,859	22/07/2014
AID 434955	323,578	28/07/2014
AID 2314	2,964,564	10/09/2015

The technical specification of GPU hardware used in the experimentation can be found in Table 2.

**Table 2:** Hardware configuration used

Particulars	GPU1	GPU2
GPU	NVIDIA GeForce GTX 780	TESLA K20
CUDA cores	2304	2496
GPU clock speed	941 MHz	706 MHz
Graphic memory	3072 MB	4800 MB
Memory bandwidth	288.4 GB/S	208 GB/S
Peak performance	4 TFlops	3.52 TFlops
Compute capability	3.5	3.5
CPU	Intel Core i7	Xeon 2650

### Interpretation

A Random Forest consists of a certain number of decision trees. Their nodes split data according to randomly selected features. This offers a better method to split features apart from the split in data, thus enabling the classifier to pick a combination of subtle changes in certain features of the data.

Tables 3 and 4 show the comparison of serial and GPU implementation of random forest on the basis of precision, recall, accuracy, ROC area and F-score on different bioassay datasets. It can be concluded that the machine learning metrics of Random Forest are not degraded when ported to the GPU. Random Forest Classifier from scikit-learn [23] was used to develop the serial version of virtual screening. The GPU version of the virtual screening was developed in Python using PyCUDA libraries [30, 31].

**Table 3:** Performance of random forest virtual screening on serial environment

Dataset	Recall	Precision	F-score	ROC area	Accuracy
AID 1332	0.68	0.5	0.58	0.73	0.89
AID 492952	0.68	0.87	0.76	0.65	0.68
AID 651616	0.77	0.95	0.85	0.49	0.74
AID 2330	0.58	0.37	0.45	0.67	0.93
AID 893	0.73	0.49	0.59	0.74	0.94
AID 778	0.49	0.34	0.40	0.62	0.78

**Table 4:** Performance of random forest virtual screening on GPU

Dataset	Recall	Precision	F-score	ROC area	Accuracy
AID 1332	0.74	0.52	0.61	0.75	0.92
AID 492952	0.73	0.87	0.8	0.66	0.72
AID 651616	0.78	0.93	0.85	0.65	0.74
AID 2330	0.54	0.38	0.45	0.68	0.93
AID 893	0.72	0.49	0.58	0.73	0.94
AID 778	0.48	0.34	0.4	0.62	0.78

Table 5 shows the depth-breadth crossover analysis for the AID2314 training set, for different values of crossover point. With the number of descriptors being constant, the optimal depth-breadth crossover largely depends on the size of the training data set. Loading small training data sets would build depth first driven trees while loading large data sets would build breadth first driven trees. Therefore, depth-breadth crossover was studied over the range from 1000 to 50,000. AID2314 is a balanced training set with 37,055 active out of the 296,456 compounds present in it. It may be noted that performance matrices do not change through the depth-breadth slide in the tree construction. The optimal crossover point of AID2314 is close to 25,000. The running time of AID2314 is the best in the table. The end user can change the main parameters, such as *bfs_threshold* and *no_of_trees_in_the_forest* in *random-forest.py* file in the code base for optimal performance.

**Table 5:** Depth-bredth threshold crossover analysis for AID2314 training set

Crossover value	Running time (s)	Recall	Precision	F-score	Roc area	Accuracy
1000	10.53	0.63	0.40	0.49	0.681	0.91
5000	10.75	0.62	0.39	0.48	0.679	0.91
10,000	11.04	0.62	0.39	0.48	0.677	0.90
15,000	10.58	0.62	0.40	0.48	0.681	0.90
20,000	10.42	0.63	0.40	0.49	0.687	0.91
25,000	10.07	0.62	0.39	0.48	0.681	0.90
30,000	10.37	0.62	0.39	0.48	0.681	0.91
40,000	10.73	0.62	0.40	0.48	0.685	0.91
50,000	12.43	0.62	0.39	0.48	0.682	0.91

**Fig. 2:**
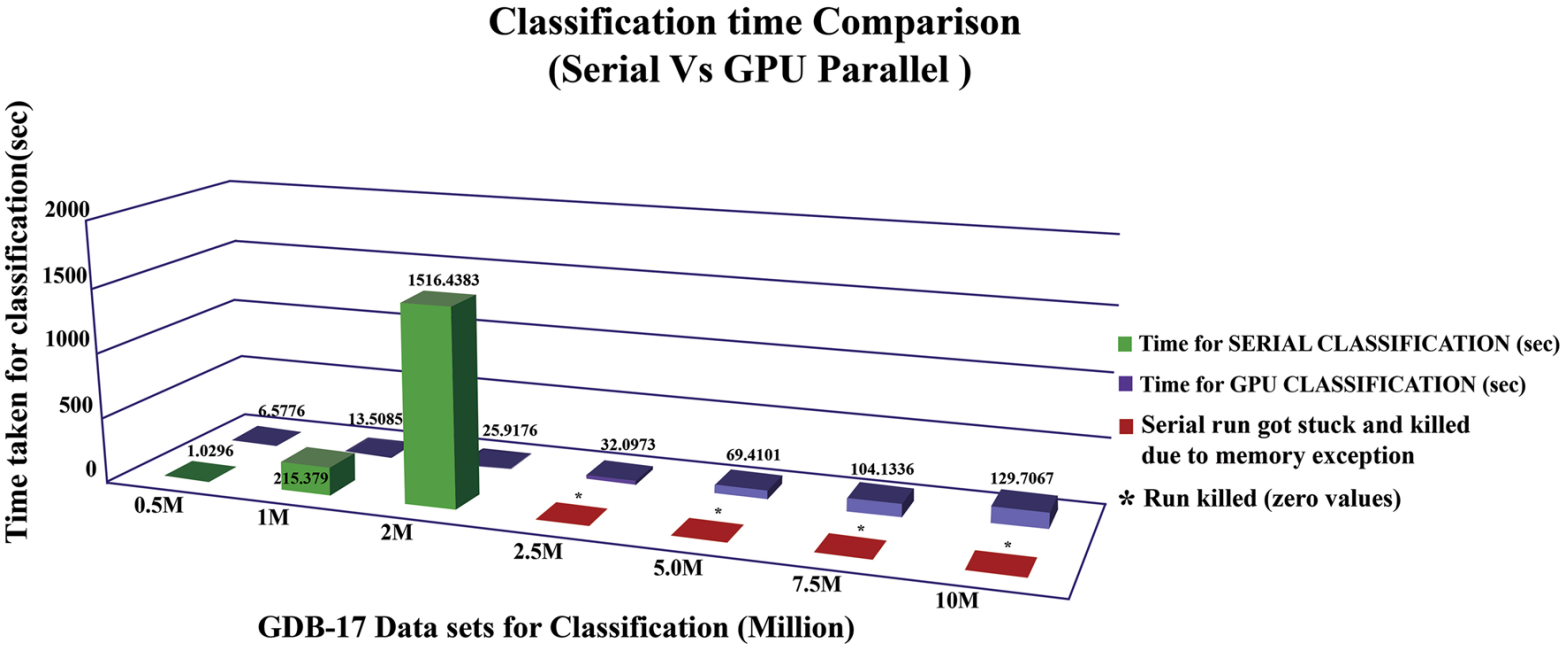
Classification time comparison: serial versus GPU

Table 6 shows the performance comparison between the training phase of the serial and that of GPU versions of RF based virtual screening on different ligand data models generated from the corresponding bioassay data in NCBI PubChem. Though there is a visible performance gain while using GPU, a major boost in performance is seen in the classification phase. The comparison of running times of the classification phase is shown in Table 7 (also see Fig. 2). For small input size, the performance gain was offset by the cost of copying the data to the GPU memory. The GPU version of random forest guarantees an increase in execution speed by 2–20 times. The speed up of the implementation increases with the number of molecules. The growth rate of execution time corresponding to increasing input size is lower for the proposed parallel implementation than the serial version. The proposed tool can easily take up billions of molecules for classification. Due to the difficulty in feature extraction of large input data, the table size is limited to ten million.

**Table 6:** Running time of serial and GPU versions of random forest virtual screening for training

Dataset	No of molecules	Time for (s) serial	Time for (s) for GPU
AID 1332	1193	0.0689	1.114
AID 492952	2294	0.2095	1.2054
AID 651616	5569	0.6641	1.7984
AID 2330	36,869	4.2428	2.5487
AID 893	68,532	11.5306	3.2746
AID 778	95,859	35.8603	9.9027
AID 434955	323,578	81.323	10.44
AID 2314	330,664	100.57	10.77

**Table 7:** Running time of serial and GPU versions of random forest virtual screening for classification

Dataset	No of molecules	Date of access	Time (s) for serial	Time (s) for GPU
Gdb 17–0.5 million	0.5 million	12/11/2014	1.0296	6.5776
Gdb 17–1 million	1 million	12/11/2014	215.379	13.5085
Gdb 17–2 million	2 million	12/11/2014	1516.4383	25.9176
Gdb 17–2.5 million	2.5 million	12/11/2014	*	32.0973
Gdb 17–5 million	5 million	12/11/2014	*	69.4101
Gdb 17–7.5 million	7.5 million	12/11/2014	*	104.1336
Gdb 17–10 million	10 million	12/11/2014	*	129.7067

* Serial exception error

The number of molecules that can be simultaneously classified in the serial environment is constrained by the amount of memory available in the machine. It is evident that this new parallel tool GPURFSCREEN outperforms the serial versions in terms of the number of molecules considered for training and classification. This parallel implementation has successfully trained more than three hundred thousand molecules on a single batch. This can be extended to up to one million in a single batch. The larger the video RAM available on the GPU, greater the number of molecules that can be trained. It should be noted that this implementation can take up billions of molecules for screening, by using the technique of partitioning the data into batches.

As evident from the results, a huge performance gain is achieved in both training and prediction phases of the learning algorithm. This contributes to a significant improvement in virtual screening of ligand based data models. The performance of the random forest classifier can be further improved by increasing the number of decision trees in the ensemble. The performance of the classifier algorithm improves with an increase in the number of decision trees steadily up to a point, after which the performance starts to decline. This optimal point of the number of decision trees can be obtained by observing the error rate. The global minima of error rate with the change in the number of decision trees may be taken as the optimal number of decision trees. However, this number is specific to each ligand data set and cannot be specified beforehand.

## Conclusion

A tool named GPURFSCREEN was developed for virtual screening process using Random Forest technique that works on a CUDA based GPU environment. Considering the large volume of data involved in ligand based drug design, this parallelized version of virtual screening is favorable for two significant reasons: reduced running time and high throughput. The computational performance offered by the GPU outperforms a multi-core system. Also, the cost of installation, power consumption and maintenance of a GPU based system are lower compared to other multi-core systems. Thus, the GPU based virtual screening for ligand based data sets is a viable alternative for quickly screening large quantities of ligand data at a comparatively lower cost.

A computational boost of 2–20 folds for Random Forest training and prediction is achieved on mediocre GPUs with a moderate number of GPU cores and video RAM. GPUs with a large number of computational cores and larger video RAM can run large bioassay data sets with significantly lower execution time. As a future extension, the virtual screening of ligand data sets can be further implemented and tested with other variants of random forest classifiers that implement balanced decision trees. The GPU implementation can also be extended to work with balanced decision trees for classification.

## Availability and requirements

- Name of tool: GPURFSCREEN
- Tool home page: Source code available at http://ccc.nitc.ac.in/project/GPURFSCREEN
- Operating system: Linux Ubuntu 13.10
- Programming language: Python
- Frame work: CUDA 6.0, PyCUDA.

## References

[1] Ekinsy S, Mestres J, Testa B (2007). In silico pharmacology for drug discovery: methods for virtual ligand screening and profiling. Br J Pharmacol.

[2] Gertrudes J, Maltarollo V, Silva R, Oliveira P, Honório K, da Silva A (2012). Machine learning techniques and drug design. Curr Med Chem.

[3] Senanayake U, Prabuddha R, Ragel R (2013). Machine learning based search space optimisation for drug discovery. Proc IEEE Symp Comput Intell Bioinform Comput Biol.

[4] Schierz AC (2009). Virtual screening of bioassay data. J Cheminform.

[5] Kirk DB, Hwu WW (2009). Programming massively parallel processors: a hands-on approach.

[6] Burbidge R, Trotter M, Buxton B, Holden S (2001). Drug design by machine learning: support vector machines for pharmaceutical data analysis. Comput Chem.

[7] Svetnik V, Liaw A, Tong C, Culberson JC, Sheridan RP, Feuston BP (2003). Random forest: a classification and regression tool for compound classification and QSAR modeling. J Chem Inf Comput Sci.

[8] Boulesteix AL, Janitza S, Kruppa J, König IR (2012). Overview of random forest methodology and practical guidance with emphasis on computational biology and bioinformatics. WIREs Data Min Knowl Discov.

[9] Alpaydin E (2003). Introduction to machine learning.

[10] Mitchell T (1997). Machine learning.

[11] Hastie T, Tibshirani R, Friedman J (2008). The elements of statistical learning data mining, inference and prediction statistics.

[12] Chen B, Harrison RF, Papadatos G, Willett P, Wood DJ, Lewell XQ, Greenidge P, Stiefl N (2007). Evaluation of machine-learning methods for ligand-based virtual screening. J Comput Aided Mol Des.

[13] Breiman L (2001). Random forests. Mach Learn.

[14] Amado N, Gama J, Silva F (2001). Parallel implementation of decision tree learning algorithms. Prog Artif Intell.

[15] Sharp T (2008). Implementing decision trees and forests on a GPU. Comput Vis ECCV.

[16] Nasridinov A, Lee Y, Park YH (2014). Decision tree construction on GPU: ubiquitous parallel computing approach. Computing.

[17] Grahn H, Lavesson N, Lapajne M, Slat D (2011) CudaRF: a CUDA-based implementation of random forests. In: Proceedings of 9th IEEE/ACS international conference on computer systems and applications (AICCSA), pp 95–101

[18] Essen BV, Macaraeg C, Gokhale M, Prenger R (2012) Accelerating a random forest classifier: multi-core, GP-GPU, or FPGA? In: IEEE international symposium on field-programmable custom computing machines vol 12, pp 232–239

[19] Liao Y, Rubinsteyn A, Power R, Li J (2013) Learning random forests on the GPU. New York University, Department of Computer Science

[20] Jenkins J, Arkatkar I, Owens JD, Choudhary A, Samatova NF (2011) Lessons learned from exploring the backtracking paradigm on the GPU. In: Proceedings of 17th parallel processing international conference, Euro-Par 2011, Bordeaux, France, vol 6853, pp 425–434

[21] Kufrin R (1997). Decision trees on parallel processors. Mach Intell Pattern Recognit.

[22] Srivastava A, Han EH, Kumar V, Singh V (1998) Parallel formulations of decision-tree classification algorithms. In: Proceedings of 27nd international conference on parallel processing, pp 237–244

[23] Scikit-learn machine learning library. http://scikit-learn.org/

[24] NCBI PubChem. https://pubchem.ncbi.nlm.nih.gov/

[25] Chemistry Development Kit. http://cdk.sourceforge.net

[26] PowerMv Molecular Viewer. http://nisla05.niss.org/PowerMV/

[27] Liu K, Feng J, Brooks A, Young SS (2005). PowerMV: a software environment for molecular viewing, descriptor generation, data analysis and hit evaluation. J Chem Inf Model.

[28] Karelson M, Lobanov VS, Katrizky AR (1996). Quantum-chemical descriptors in QSAR/QSPR studies. Br J Pharmacol.

[29] Lars R, van Deursen R, Blum LC, Reymond JL (2012). Enumeration of 166 billion organic small molecules in the chemical universe database GDB-17. J Chem Inf Model.

[30] Klockner A, Pinto N, Lee Y, Catanzaro B, Ivanov P, Fasih A (2012). PyCUDA and PyOpenCL: a scripting-based approach to GPU run-time code generation. Parallel Comput.

[31] Sanders J, Kandrot E (2011). CUDA by example: an introduction to general purpose GPU programming.

